# Remarks on Appendicitis, with a Report on Twenty-six Cases Operated on during the past Twelve Months

**Published:** 1897-05

**Authors:** Hunter McGuire

**Affiliations:** Professor Clinical Surgery University College of Medicine, Richmond, Va.


### REMARKS ON APPENDICITIS WITH A REPORT OF TWENTY-SIX CASES OPERATED ON DURING THE PAST TWELVE MONTHS.

BY HUNTER McGUIRE, M. D., LL. D.,

Professor Clinical Surgery University College of Medicine, Richmond, Va.

*Mr. President and Gentlemen of the Medical Society of Georgia:*

First, let me thank you for the kind invitation of your distinguished president, which gives me the pleasure of being with you to-day, and the honor of reading a paper before your Association.

For a time I was at a loss to know what subject to select, but finally concluded that it might prove of interest to report my work in appendicitis for the past year, and to discuss a few practical points connected with the disease. An analysis of the twenty-six cases reported will show that nineteen were of the chronic form, and seven of the acute; that thirteen occurred in males and thirteen in females; that twenty-five recovered and one died.*

So much has been written on appendicitis that I do not propose to discuss the subject systematically, but simply wish to give my views briefly on several questions in which there are still legitimate differences of opinion, and thus invite a general discussion of the subject on the part of the society.

### I. THE IMPORTANCE OF OPERATING FOR APPENDICITIS DURING AN INTERVAL BETWEEN ATTACKS.

All of the nineteen cases of chronic, or relapsing, appendicitis embraced in the present report recovered. In looking over my record book for previous years I find that I have operated on thirty-three other cases with only one fatal result, thus making a total of fifty-two consecutive operations for this form of appendicitis, with one death. It is only fair to add that one of my cases was operated on for me by Dr. Joseph Price, who generously came from Philadelphia to do the work which, for some personal reasons, my son, Dr. Stuart McGuire, and I wished to avoid. The results I have attained are no better than many other operators have had, and I attribute my success largely to the fact that, if possible, I operate during the quiescent stage, as Mr. Treves originally suggested in 1886, when danger of sepsis is passed and inflammatory symptoms have disappeared. The danger is practically nothing if the surgery is clean. If you will carefully read some of the reports of my cases you will see that I sometimes wait, and possibly in a few instances with some risk to

*In all these operations I have been assisted by my son, Dr. Stuart McGuire, who in many of the cases did most of the work, Dr. E. L. Hobson gave the chloroform; Dr. Frank Hancock, of St. Luke's Home, and Drs. Edwards and Wooling, of the Virgina Hospital, were also assistants.

the patient, until the acute symptoms pass off and sufficient time has elapsed for all inflammation to disappear. This requires generally two or three weeks, and during this period of waiting I keep the bowels freely open, attend carefully to the diet, restrict exercise by confinement to room or bed, and give 1-30 of a grain of bi-chloride of mercury three or four times a day as an intestinal antiseptic. In this way I have been able to tide the patient over the acute stage in a number of cases, and to operate during the interval between the attacks.

### II. Symptoms Which Determine the Safety of Postponing Operative Interference until the Acute Stage has Passed.

When we remember that fully one-half of all the cases of appendicitis recover from the first attack; that spontaneous resolution takes place when peritonitis, exudation, and sometimes even suppuration, have been present; and that a large number of cases which recover without an operation in the primary attack remain well and have no recurrence of the disease, the question of when to operate or not to operate is a very nice, difficult, and always an important one.

I agree with Wyeth, that if you have a competent surgeon, one skilled in abdominal work, and the patient can be operated on within twelve hours of the first symptoms of the attack, ninety-nine per cent. will be cured. But we frequently see the cases after these twelve hours have expired, and surgeons of experience in such work are not always within call. I confess I am not in favor of an operation in all cases as soon as a positive diagnosis is made. Too many of them get well and stay well, to warrant it, and others can be made to wait, and have the operation done during an interval between the attacks, when the inflammatory symptoms have almost if not quite disappeared, the danger of sepsis has been passed, and the risk of death from an operation is almost practically *nil*.

When the attack is sudden and severe, the pain intense, pulse rapid, temperature high, respiration short and thoracic, abdomen hard and distended, face pinched and anxious—especially if the group of symptoms have been ushered in with a chill—the only hope for life is an operation, and the sooner the better. Such a case is called a fulminating one (it comes on like an explosion), and we are not warranted in waiting an hour to try medicinal remedies. In such cases if, along with other symptoms, there is shock, perforation will almost certainly be found, and the chances for life are very slight even with an early operation. Fortunately such cases are very uncommon, but it is just such cases that have made some good surgeons say, "Operate in all cases, and at once, when the diagnosis is made." It is these fulminating cases that have produced a panic among the lay people, and have struck terror among many practitioners of medicine. I have seen during the past year but one case of perforating appendicitis; the man was moribund when I was called in and no operation was attempted.

When the symptoms are presented more slowly and less severely, when the pain is not so great, abdomen flat, pulse, temperature and respiration nearly normal, little or no swelling or hardness in the right iliac region, then there is no such reason for haste. We can safely wait for some hours and watch the progress of the case, and this watch should be most carefully made. Of all the cardinal symptoms the temperature is the least reliable, and, in many cases, should be the least considered. The nausea and vomiting, and the effect of the disease on the sympathetic system, is often so profound as to keep the temperature down to almost normal. In cases Nos. 17 and 25 the temperature was 99°, and yet I found in each the appendix dead, and so rotten that it was difficult to handle it without tearing it; but it is important to remember that while the absence of high temperature is no indication that serious mischief is not going on, the presence of a high temperature, say 102°, shows that serious changes are probably taking place. The most important symptom to note is the pulse; it is what we used to call the "abdominal pulse," small, thready and frequent. As the case improves the pulse gets less frequent and less small, and *vice versa*, when the inflammation grows worse the pulse gets more frequent. In appendicitis a pulse of 120° is a sign of great danger. When the pain grows worse, the pulse faster, and possibly the temperature higher, and rigidity of muscles over the inflamed spot greater, the case is growing worse.

### III. VALUE AND LIMITS OF SALINE PURGATION.

In an ordinary case, not a fulmintaing one, after a short time the pain abates, the fever lessens, the pulse gets slower, the abdomen less hard and tender, and all the urgent symptoms more or less subside—they do not disappear, but are less pronounced—and then there comes in a remedy which in my hands has been as certainly valuable for good as quinine in malaria or arsenic in neuralgia; that is saline purgatives, given in frequent doses until the patient is freely and repeatedly purged. The copious and numerous watery operations "bleed" the bowel and peritoneum and lessen the congestion or inflammation. I have watched case after case during this period of lull in the symptoms, in grave doubt whether the exudate would be taken up and resolution of the inflammatory product occur, or whether it would continue on to suppuration or gangrene, or both, and have seen the former rapidly follow free saline purgation.

If the disease continues after the purging, the abdomen is left in a better condition for a future surgical operation. I am always glad, in any abdominal or pelvic surgery, to know that my patient has been well purged and that the bowels are empty. If, after free purging, the pain continues, the pulse is rapid, the belly tender, an operation should be done without further delay. Some surgeons are strongly opposed to the attempt to purge, and in some cases rightly so. In fulminating appendicitis, an attempt to purge would most likely fail and the purgative add materially to the trouble; and to try to purge a case when pus or gangrene was present would be simply silly and mischievous; but to denounce saline purgation in every case is as wrong in principle and in fact as to demand operation in every case as soon as the diagnosis of the disease is made.

IV. Relative Frequency of the Disease in the Two Sexes.

From the reports of many surgeons we learn that appedicitis is much more common in males than in females—the proportion stated is one to four. This has not, however, been my experience. In the cases for the past year, which I present today, it will be seen that thirteen were females and thirteen were males, and this has been about the proportion of all my cases, amounting now to one hundred and fifty-five, the cases being nearly equally divided between the two sexes. At one time, say ten years ago, it was believed that the female was almost exempt from inflammation of the vermiform appendix. Lately, however, more and more cases have been seen in that sex. There is certainly no difference in the anatomy, physiology, or pathology of the organ in the two sexes to account for its greater frequency in the male; the additional little artery—sometimes but not always found in the female, and supposed to give better nutrition to the appendix in that sex—will scarcely explain the difference. By some it is ascribed to injury to the appendix by the psoas muscle in the male. Is the relation of this same muscle in the female so markedly different? I think not.

Frankly, I am at a loss to know how to explain my record. I know that I am no better and, in many cases, not so good, a diagnostician as some other surgeons, and yet I can not help sometimes thinking that the very aggressive gynecologists, and I beg to assure any of them present that I mean no offense, have taught us so much about ovaritis, salpingitis, tubal pregnancy, and so forth *ad infinitum*, that the hard-working honest practitioner is led to believe that every unusual symptom of trouble about the pelvis must refer to some one or other of the female organs of generation, and I fear many a case of trouble about the cæcum and appendix has been treated for ovarian or tubular disease. Indeed, I confess to having made the mistake in more than one instance myself. In one of the cases in this report, in my ignorance I treated the patient for some months for ovarian trouble, and when at last the symptoms were so pronounced that I could no longer mistake them and I operated, I found an adherent appendix filled with pus. I think it would be well, when called in to see an obscure case of pelvic trouble in the female, not to let the gynecologist make us believe that there is nothing else in a woman's pelvis but the uterus, tubes and ovaries. I recall a case, fifteen years ago, of a lady to whom I was called in consultation by Dr. Lewis Wheat, of my city. Two days before she had been delivered of a child and was supposed to be suffering from puerperal sepsis. She died and, as the labor had been easy and natural, it was difficult to explain the result. Dr. Wheat made a post-mortem and found that a large abscess of the appendix had bursted during labor and flooded the cavity of the abdomen with pus. In this connection Case No. 21 is interesting. This lady, married, aged 33, had been in bed for five months, suffering from frequent attacks of violent pain in the right iliac region, spreading over the whole of the abdomen. For these attacks she was given morphia by a physiciau then in attendance, but now dead. The attacks were so frequent and the morphia given so liberally that she became an opium *habitue* and was miserable without the drug. She was naturally a weak, hysterical woman, and this state of mind and body, along with the opium, made it very difficult to get from her a true story of the case. I found that she was pregnant about four months (although she denied it most emphatically), and that her tubes and ovaries were free from disease. She was tender over the right iliac region, and I found there more muscular tension than upon the opposite side; there was no swelling. The point of tenderness was in the right flank on a line with the anterior superior spinous process, and not in the pelvis. An examination through the vagina of the contents of her pelvis gave her no pain. When the attacks were at their worst she had vomiting and tympanitis. When I opened the abdomen I found an inflamed and adherent appendix, with some exudate but no pus. She got well, gave up voluntarily the morphia before she left the hospital, and is now in the eighth month of pregnancy. This is the second time I have operated for appendicitis in pregnant women—the other case at the sixth month of pregnancy. Both cases recovered and neither miscarried. I do not think one should hesitate to operate during any period of pregnancy, or even during parturition, if the case required it.

### V. DIFFERENTIAL DIAGNOSIS BETWEEN APPENDICITIS AND DISEASE OF THE TUBES AND OVARIES.

The diagnosis is not easy when inflammation of the right tube and ovary and of the appendix occur at the same time as in Cases Nos. 9, 13, 14 and 20. We have in both rapid pulse, rise of temperature, pain, vomiting and tympanitis. There are, however, some points of difference that will often enable us to distinguish between the two diseases. Appendicitis begins more acutely. If it is a chronic case there is a history of one or more former sharp and sudden attacks. Lesions of tubes and ovaries are of older date and have a history of menstrual disorder. The pain of appendicitis is acute, frequently violent, beginning over the solar plexus, radiating over the whole belly and finally settling in the right iliac region. In adnexal disease the pain is dull and heavy, and never sharp and lancinating until the peritoneum is involved. The patient is more alarmed in appendicitis than in disease of the adnexa. The location of tenderness is different; in appendicitis it is on a level with the anterior superior spine; in adnexa trouble it is in the pelvis. In the latter, vaginal examination reveals the site of tenderness; in the former, you can touch and move the organs in the pelvis without producing pain. Vomiting is more common in appendicitis. Rigidity of the muscles of the abdominal wall over the right iliac region is almost always present in appendicitis, and generally absent in inflammation of the tubes and ovaries. Indeed, the muscular rigidity and location of the tenderness, or pain, will usually decide the diagnosis, but in case of doubt I would give chloroform and by its aid the enlarged and tortuous appendix can be felt, probably fastened in its place by adhesions; or, by a bi-manual examination, we may discover disease of the adnexa. In cases of pus-tube on right side I believe the appendix, whether diseased or not, should be removed when the pus-tube is taken away.

### VI. TECHNIQUE OF OPERATION.

*In fulminating cases* the abdomen should be opened by a long incision, the gangrenous appendix ligated and cut off, and the belly washed out with gallons of sterilized water. Six or eight strips of iodoform gauze should be carried through the incision to different parts of the cavity. The wound should not be sutured, but every facility afforded for easy drainage. If such a case is not seen until the bowels are distended with gas, and the patient prostrated or collapsed, it is useless to operate. I have never seen a case of fulminating appendicitis without premonitory symptoms. A careful inquiry into the past history will always show former attacks which, possibly, however, at the time were not recognized.

*In cases where there is a deep-seated localized abscess* it should be rendered accessible by a free abdominal incision. The abscess should be carefully walled off from adjacent tissues by pieces of gauze and then opened. The pus should be sponged out and the appendix carefully searched for and removed. The abscess wall should be dissected out and the bowels examined to see that they are intact. It is impossible to treat such cases extra-peritoneally, and when you have exposed the cavity to the danger of infection there is no more excuse for doing incomplete work than in other forms of abdominal surgery. Such a case was No. 12.

*In cases where the abscess has approached the anterior abdominal wall and became adherent to the peritoneum* I simply open and drain it, and make no effort to extract the appendix if not loose, or to remove the wall. To endanger the infection of the general cavity by too prolonged an attempt to find the appendix is not, I believe, good surgery. The temptation is sometimes right great to persist in efforts to get it, as the patient and his friends feel a sense of disappointment when told that the appendix was not found. The disappointment is almost as great as they would have when cut for stone in the bladder and told that the stone could not be found. But no amount of anxiety on their part will justify the surgeon's running the risk of bursting through the wall and exposing the cavity to infection. If the wall is broken from any cause then, after sponging out all of the pus, I would remove it as you would do in case of pus-tubes. This was done in Cases No. 12 and No. 22.

*In cases of chronic or relapsing appendicitis* I do not advise an operation until the patient has had two attacks. Sometimes the inflammation which attends the first attack renders the patient free from a second attack because of some obliterating pathological change in the appendix. I make one exception to this rule—if, several weeks after the first attack I find tenderness or induration, or both, over the region of the cæcum, I advise the patient to have the operation done at once. The operation should always be done between the attacks. In every case all adhesions should be freed, and any portion of the omentum in contact with the appendix which is suspected of being contaminated, should be removed.

---

CASE I.—Mr N. W. C., Richmond, entered St. Luke's March 2, 1896. Has had four attacks of appendicitis. He is a traveling salesman, and has been taken on each occasion while traveling in a railway car. Because of persistent tenderness in the right iliac region, and fear of an attack when out of reach of a surgeon, he asked to have the appendix removed.

The appendix was found adherent to the cæcum, and fastened like a strap over the bowel; a piece of omentum was adherent to it. The adhesions were undone after much trouble, and the appendix removed. No drainage. Made a good recovery.

CASE II.—Miss G. M., Richmond, has had repeated attacks of appendicular colic. The pain in all the attacks has been very violent, and on each occasion required the use of morphia. In the last seizure, morphia failed to give relief, and I resorted to chloroform, keeping her under its inflence for several hours. As there was good reason why neither my son nor I should operate in this case, Dr. Joseph Price, of Philadelphia, kindly performed it for me in St. Luke's Hospital. The appendix was easily found and removed. There were no adhesions. The organ was inflamed and thickened and at one or more points its lumen contracted. Completely recovered.

CASE III.—Miss R. B. W., *æt* 19, of Orange county, Virginia, patient of Dr. Rowe. History of two severe attacks of appendicitis; the last one confined her to her bed for several weeks. Never has been free from pain since last attack; had constant tenderness, with slight exacerbations of almost daily recurrence; can not stand up straight without a sense of something pulling her down.

The appendix was adherent by its tip to the cæcum, and sharply bent on itself. Removed it in the usual way. Recovered without any interruption.

CASE IV.—Mr. X., of Gayton, Virginia, admitted to the Virginia Hospital March 18, 1896; patient of Dr. R. T. Davis. Two weeks ago recovered from his third attack of appendicitis; the last attack attended by pain, fever, swelling, some tympanitis; was in bed two weeks.

Operated before the class; found the appendix tortuous, enlarged, and filled with foul-smelling pus. No drainage; speedy recovery.

CASE V.—Mrs. J., of North Carolina; patient of Dr. Sorrell; entered St. Luke's March, 1896. Has been an invalid for several years, with inflammation of the adnexa. Along with the constant pain accompanying this condition, has had many violent attacks of severe pain, fever, tymyanitis, swelling in the right iliac regoin, vomiting, etc. Diagnosis: pus-tubes and chronic appendicitis. Took large quantities of morphia, partly for the pain and partly from habit. Found pus-tubes on both sides, which were shelled out with some trouble. The appendix was six inches long, attached to right tube. A piece of omentum was attached to the appendix. This last was detached, tied, and cut off. The appendix was removed. Drainage with gauze for three days. Gave her ¼ gr. morphia every three hours first day after the operation and gradually lengthened this interval. She recovered without much trouble, is well, aud free from the opium habit.

CASE VI.—Miss N. B. C., aged 17, came from a large boarding-school in Staunton, Virginia, and entered St. Luke's March, 1896. Her physician, Dr. Atkinson, diagnosed appendicitis. When she arrived in the hospital the right flank was hard and swollen, the legs drawn up, pulse rapid, temperature 104½°. Her nervous condition was so bad, and she looked so like dying, that I ordered turpentine enemas, opium, and hotwater cloths to her belly. She improved at once after her bowels were emptied. Day after day she got better, until in a week she was almost free from pain, swelling, and fever. In the iliac region there was still tenderness and hardness. After she had been in the hospital a week I operated, and found only a few fragments of the appendix, which I scraped away. The cæcum was tightly fastened to the iliac fossa, and was loosened with difficulty. Under the cæcum was found the abscess. It was cleansed, curetted, and drained with iodoform gauze. Her recovery was slow, but perfect.

CASE VII.—Mr. A. B., aged 25; admitted into the Virginia Hospital March 29, 1896. Has had some marked attacks of appendicitis, the last one month ago. Besides the severe pronounced attacks, he has had several minor spells. While quiet, he is free from suffering, but any attempt at exercise gives pain. The tenderness on pressure is constant.

Operation before the class. Found stricture near the cæcum, lumen entirely occluded, and distal end filled with pus. No adhesions of any note, no drainage; speedy recovery.

CASE VIII.—Mr. W. W. B., of Charleston, W. Virginia, railway conductor, was admitted to St. Luke's Home March 8th, 1896. He has had two violent attacks of appendicitis at tended with pain. Both attacks came on very suddenly when he was on his way from his boarding-house to take charge of his train. The attacks were attended by great pain, requiring free use of morphia. He had high fever in both cases. The tenderness in the right iliac region still continues. I find on palpation a well-marked induration in the iliac region with great tenderness. I operated on him March 12th; found the appendix very large and long; down in the pelvis and attached closely to the pelvic walls. I had to peel it out with my finger as I would a pus-tube. Indeed, it felt to my finger very much like the ordinary pus-tube. When I brought it out of the wound, there came out with it a piece of omentum fastened to it so tightly that in separating it, I ruptured the tube, and some slight amount of pus escaped. Fortunately it was then on the outside of the abdomen. The whole structure seemed to be deprived of peritoneum, so that I could not make a flap of the serous membrane to cover the stump. I tied it off and applied very freely to its exposed surface the thermo-cautery. Introduced some gauze drainage and closed the wound. His physician was Dr. Tinker, of Athens, Ohio. His recovery was complete.

CASE IX.—Mrs. F. S., of Richmond, Virginia, aged 37, was admitted into St. Luke's April 10, 1896. Has had attacks of pain in her right iliac region for twenty years. At one time I believed the trouble was ovarian, and treated her with electricity, the local use of iodine, and internal administration of anodynes and alteratives. Lately the attacks have resembled appendicular colic, and she insists that the pain is different in character from that she formerly had. The appendicular attacks have been coming at shorter intervals and more severe; none of them, however, attended with fever until the last attack, when she had marked fever, rigid abdominal walls and great pain, the attack lasting for a week. Indeed, she never got over the last attack, as she had constant pain and stiffness in the right iliac region. I operated on her April 10th; found the appendix fastened to the omentum and to the right broad ligament. Its attachments were strong, and I had to dig them out with the blunt end of a pair of forceps. When removed, the lumen of the appendix was found occluded about half way from its tip to the cæcum, and contained in its free end half a drachm or more of bad-smelling pus. The right Fallopian tube was large, inflamed, and red, and the ovary had undergone some cystic degeneration. The tube and ovary on that side were removed. The left tube and ovary were sound; no drainage; recovery uninterrupted.

CASE X.—Mrs. C., aged 30, admitted to Virginia Hosital March 10, 1896, has had repeated attacks of appendicitis. The last one kept her in bed for six weeks. Operation done before the class; appendix enlarged and thickened and fastened to cæcum; made two holes in the cæcum in dissecting it away, which were closed with fine silk sutures. Appendix contained two hard concretions like cherry stones, but were really composed as usual of fæcal matter. No drainage; recovery uneventful.

CASE XI.—Mr. S. S. D., of Macon, Ga., aged 20, was admitted into St. Luke's Home April 25, 1896. This gentleman had a violent attack of appendicitis eighteen months ago while in London, with peritiphlitis, which kept him in bed for two or three months. He was exceedingly ill there, and at one time his physician thought he would die, but he improved sufficiently to be put on the steamer and brought home. About eight months afterwards he had a second attack at Macon, Ga., about as severe as the first. Since that time he has had repeated attacks of pain in the region of the cæcum, necessitating sharp purging and occasional application of leeches, poultices, etc. He came to see me six months ago, and was so entirely free from any remnants of the former attacks that I decided to let him alone and sent him back home. After he returned home, however, he had several pretty sharp attacks, and came back. After reaching Richmond he stayed with his sister, and again for a month was free from any soreness in that region. A day or two after, however, great soreness came on, and his suffering was very severe. I opened his abdomen April 25th, making the incision over the region of the cæcum. Found the cæcum intimately adherent to the iliac fossa and evidence of great inflammatory deposit there.

The ilium for several inches was fastened to the cæcum and attached by bands to the posterior abdominal wall. All these attachments were broken up and the appendix was found in its usual site, but nothing but the stump was left. No trace of it except this little projection of about half an inch could be found. At this writing, May 1st, he has not had a bad symptom. The operation occupied one hour and twenty-five minutes. His physician was Dr. Mettauer, of Macon, Ga.

CASE XII.—Mr. D. P. S., of Staunton, Virginia, aged 38, was admitted into Saint Luke's Home May 20, 1896. Acute appendicitis. Was taken ill on May 16, 1896. Suffered terribly on the 17th, 18th, and 19th. Came to me on the 20th saturated with morphia, of which he had taken frequent hypodermics. Fortunately, he had been pretty well purged. He had all the symptoms of the formation of an abscess of the appendix.

I operated that night; found the appendix gangrenous and sloughing, and a large abscess, which I peeled out as you would do a common pus-tube; sprinkled iodoform thickly in the site of the abscess cavity, and drained with both glass tube and gauze. His recovery was slow, but complete.

CASE XIII .—Mrs. O. S., of Richmond, Virginia, aged 20, was admitted to the Virginia Hospital October 10, 1896. She had been an invalid since the birth of a child some months previous, and suffered pain over the region of both ovaries, that on the right side being the most constant and acute.

The abdomen was opened to remove the diseased organs, and the appendix was also found to be involved. It was thickened, inflamed, and adherent to the right Fallopian tube. The tubes, ovaries, and appendix were all removed, and the patient made a quick recovery, and has since remained free from symptoms.

CASE XIV.—Miss K. R., of Richmond, Virginia, aged 28, was admitted to the Virginia Hospital November 28, 1896. History of gonorrhea, and several attacks of peritonitis. Ovaries tender, and tubes as large as sausages.

When the belly was opened, and numerous adhesions separated, the appendix was found fastened to the right ovary and buried in a mass of lymph. It was removed, along with the diseased adnexa, and found filled with muco-pus. Prompt recovery.

CASE XV.—Mrs. W. R., of Looney, Va., aged 40, patient of Dr. Givens, was admitted to the Virginia Hospital December 19, 1896. She had an ovarian tumor, which distended the abdomen to the size of a women pregnant six months. An operation for the removal of the cyst found the appendix firmly adherent to its walls, and distended with muco-purulent contents. The tumor and appendix were both taken out, and the woman recovered, and went home well.

Case XVI.—Mr. I. J. M., of Richmond, Va., aged 30, was admitted to St. Luke's Home January 6, 1897. He has had repeated attacks of appendicitis, some of them violent, and attended with fever. The last one was about four weeks ago, and the most severe one he had had.

I opened the abdomen; found the cæcum and appendix bound down with innumerable adhesions. I had to dig out the appendix with my fingers, and sometimes to use the scissors and knife. Pus found in it. He did not do well after the operation. He took chloroform so badly that at one time I had to raise the window and give him fresh air. The weather was cold, and he took cold on the table, and for a few days was threatened with pneumonia. He had a violent, persistent cough, jerking in this way at the stitches, so that every stitch-hole suppurated. In this way the healing was delayed. His recovery was slow, but complete.

Case XVII.—Mr. J. L., of Richmond, Va., aged 35, was admitted to St. Luke's Home January 6, 1897. Has had repeated attacks of appendicitis, without knowing what the trouble was. He is a patient of Dr. William S. Gordon, who called me to see him in consultation on the night of the 5th of January. From the patient's report and that of his wife that night, he seemed to be better than he was the day before. He is a phlegmatic individual, and difficult to get any information out of, so Dr. Gordon and I decided to let him alone a few hours longer. In coming out of the house with my hat and overcoat on I met his stepmother, who told us that he had a severe chill about three o'clock in the morning of that day, followed by intense fever; this succeeded by a drenching sweat, this by complete ease and sleep; information that we had not been able to get out of the patient or his wife. We then decided to operate at once. He was removed to the hospital, and the operation was at once performed.

The appendix was enlarged, and about as big as my index finger, soft, containing fæcal matter and pus, with patches here and there of mortification. I shelled it out of its bed without bursting it, and removed it in the usual way, applying the thermo-cautery to the stump, and dusting the site that the appendix had occupied in the cavity thickly with iodoform. The man was septic from the dead and dying appendix; showed it by his rather rapid pulse, yellow skin, and delirium at night. Purging, however, got him out of this, and he made a good recovery.

Case XVIII.—Mrs. M. L., of Huntington, W. Va., aged 45, was admitted to the Virginia Hospital January 9, 1897. She has had numerous attacks of relapsing appendicitis, distributed over the last fifteen years, and was thoroughly incapacitated for work. She was operated on in a public clinic, and the appendix, which was found inflamed and adherent, was removed.

She made a tardy convalescence, owing to an attack of grippe; was discharged from the hospital after six weeks, and is now well.

CASE XIX.—Mrs. J. G. D., of Tennessee, aged 21, was admitted to St. Luke's Home January 16, 1897. This lady had been married a month and was on her bridal tour through Florida when she was suddenly taken with appendicitis, and her husband brought her here.

I operated on her the evening of January 16. This was certainly the third, possibly the fourth, attack the lady had had. Found the appendix matted down by many adhesions. I removed it, however, without much trouble. It showed marks of old and recent inflammatory changes. Recovery uninterrupted.

CASE XX.—Miss J. C., of Childrey Store, Va., age 20, was admitted to the Virginia Hospital January 16, 1897. She had suffered great pain in right side for years and was bedridden.

She was operated on before the class, and the appendix and right ovary found inflamed and amalgamated. Both organs were removed, and the appendix was found constricted at its base and filled with a gelatinous material. Easy convalescence.

CASE XXI.—Mrs. N. J., Staunton, Va., aged 36, was admitted to St. Luke's Home January 21, 1897. She has been confined to bed for six months with attacks of abdominal pain. Her physician felt compelled to use morphia very freely, and she soon refused to do without its daily use. She had a child two years ago, and no special trouble at its birth. She denies being pregnant now. Complexion is like a piece of leather, is emaciated to the last degree and scarcely speaks above a whisper; lives on beef extracts and suffers almost constantly with nausea; has occasional attacks of violent pain, located usually about the umbilicus. The attacks are accompanied with vomiting and tympanitis; no more tenderness over one iliac region than other, but very marked increased muscular tension on right side.

Made the opening in the middle line, as the diagnosis was not clear; found uterus three months pregnant, tubes and ovaries sound, and appendix bent, swollen and filled with stinking pus. Recovery was uneventful. She gave up the morphia habit, and is now well and in the eighth month of pregnancy.

CASE XXII.—Master S. G., of Richmond, Va., aged 5, was admitted to St. Luke's Home January 27, 1897. This little boy was brought in by Dr. Stuart McGuire, with acute appendicitis.

Found the abscess containing about a tablespoonful of pus, and the appendix so rotten that it broke in a number of pieces on being removed. The wound was left open, no stitches used and the site of the abscess packed with iodoform gauze. He did well for twenty-four hours, then became restless and was beyond all control—crawled all about the bed—laid on his back, side or belly, as he pleased. He was purged the day after the operation by a dose of calomel. The second day we gave him one-sixteenth of a grain of morphia, which was repeated at short intervals, and he presently became quiet. Discharged February 13, well.

CASE XXIII.—Mr. L. R., of Island Ford, Va., aged 20, was admitted to St. Luke's Home February 6, 1897. He has had two severe attacks of appendicitis; the last one came on while he was in Basic City. Treated by his local doctor with opium and sent to me as the case was subsided. When he reached me I waited a few days for all inflammatory symptoms to disappear and all danger of sepsis was passed.

I opened the abdomen February 12, and found the appendix fastened down by a large number of adhesions; bowels were adherent one to the other, but the adhesions were easily unloosened and undone. The appendix was occluded, inflamed and contained a small quantity of pus. No drainage. Recovery was without interruption. His father, Dr. Richards, of Rockingham county, writes me that he is well and at work.

CASE XXIV.—Mr. H. C. W., of Lynchburg, Va., aged 25, was admitted to St. Luke's Home February 8, 1897. Has had five attacks of appendicitis; last one about three or four weeks ago. Each attack seemed to get more and more violent. Slightly tender now over the region of the appendix.

Operated on him Friday, February 12. Found the appendix adherent; had to dig it out of a mass of inflammatory deposit. The appendix was enlarged, with stricture; contained a large quantity of bloody, bad-smelling, dark-looking fluid. No drainage. His recovery was complete.

CASE XXV.—Mr. C., New Haven, Conn., aged 30, was admitted into the Virginia Hospital February 16, 1897. He is a patient of Dr. Edward McGuire; was taken ill three days ago with violent pain, tenderness and muscular rigidity in right side, tympanitis, etc. His physician purged him fully with Epsom salts and the symptoms abated. In a few hours the pain returned; pulse was rapid, face anxious, muscular tension decided.

Operated at his physician's request; found appendix swollen, enlarged, dark and gangrenous in places. It was filled with black, stinking fluid of some kind. At the time of operation temperature was 99°. So urgent was this case considered that I decided to operate at 2 P. M., and not wait for his father, who was expected to arrive in Richmond at 7 P. M., the same day. Gauze drainage was freely used, and a large part of the wound left open. His recovery was uneventful.

CASE XXVI.—Mr. G., was admitted to St. Luke's Home February 27, 1897, with an acute attack of appendicitis, which had been going on for fourteen days, I was out of the city, and the operation was deferred until the day after he arrived in the hospital.

Found on opening the abdomen such a mass of disease as I have never seen before in such cases. The appendix was not certainly found. Cut away a mass from the omentum which was supposed to contain the appendix with inflammatory deposits, but I was not sure of it; the parts were so disorganized that I could not tell. Separated a number of adhesions, and packed with iodoform gauze for drainage, but the sepsis under which he was laboring when I operated continued, and after six days he died. The death of this man was due to procrastination.

